# The diversity of fungal genome

**DOI:** 10.1186/s12575-015-0020-z

**Published:** 2015-04-02

**Authors:** Tapan Kumar Mohanta, Hanhong Bae

**Affiliations:** Department of Biotechnology, Yeungnam University, Gyeongsan, Republic of Korea

**Keywords:** Ascomycota, Basidiomycota, Chytridiomycota, Monoblepharidomycota, Neocallimastigomycota, Blastocladiomycota, Glomeromycota, Entomophthoromycota, Stramenopiles and micorsporidia

## Abstract

The genome size of an organism varies from species to species. The C-value paradox enigma is a very complex puzzle with regards to vast diversity in genome sizes in eukaryotes. Here we reported the detailed genomic information of 172 fungal species among different fungal genomes and found that fungal genomes are very diverse in nature. In fungi, the diversity of genomes varies from 8.97 Mb to 177.57 Mb. The average genome sizes of Ascomycota and Basidiomycota fungi are 36.91 and 46.48 Mb respectively. But higher genome size is observed in Oomycota (74.85 Mb) species, a lineage of fungus-like eukaryotic microorganisms. The average coding genes of Oomycota species are almost doubled than that of Acomycota and Basidiomycota fungus.

## Introduction

Fungi are the larger group of eukaryotic organisms that ranges from yeast and slime molds to mushrooms. These organisms are majorly classified as monophyletic Eumycota group and their diversity ranges from 500 thousand to 9.9 million spanning over 1 billion years of evolutionary history [1,2]. They are abundant at worldwide scale due to their small size and their cryptic lifestyle in soil, dead and decomposing matter, as symbionts with algae, fungi, bryophyte, pteridophyte, higher plants and animals [3-7]. These organisms dominate earth from polar to temperate and tropical habitats [8-10]. Due to their ecological dominance, they play a central role in human endeavor. The fungus (mushroom and truffle) are directly used as human food and yeasts are used in bread industry. The fungi also carry out nutrient cycling by decomposing organic matter [11-13]. They also produce antibiotics, enzymes, mycotoxins, alkaloids, polyketides and other chemical compounds [14-21].

The kingdom fungi are classified into several major phyla namely Ascomycota, Basidiomycota, Chytridiomycota, Monoblepharidomycota, Neocallimastigomycota, Blastocladiomycota, Glomeromycota, Entomophthoromycota, Stramenopiles and Micorsporidia and sub-phyla namely Kickxellomycotina, mucoromycotina and Zoopagomycotina [22,23]. The diverse ecological dominance of fungus makes them important from an evolutionary point of view. That is why fungi are subjected to intense phylogenetic, ecological and molecular studies. The advancement in high throughput sequencing technology progressed rapidly that led to sequencing of large numbers of fungal genomes. The evolution of biological diversity raises several questions such as how much variation can be expected among closely or related genomes. This can be answered by the comparing closely related genomes. So we carried out a global search of fungal genomes in MycoCosm and JGI database and studied the evolutionary relationships of their genome sizes and reported here [24-26].

### Fungal genome size

Recently, the genome sequencing technology has emerged as one of the most efficient tools that can provide whole information of a genome in a small period of time. Since the completion of genome sequencing of the model fungus *S. cerevisae* in 1996, sequencing of large numbers of fungal genomes are now completed. Sequencing of large numbers of fungal genomes will allow us to understand the diversity of genes encoding enzymes, and pathways that produces several novel compounds [24]. Although the fungi are very diverse in nature, their basic cellular physiology and genetics shares some common components with plants and animal cells. These include multi-cellularity, cytoskeletal structures, cell cycle, circadian rhythm, intercellular signaling, sexual reproduction, development and differentiation [27]. It was previously thought that genomes of all fungi are derived from the genome of the model fungi *Saccharomyces cerevisae* [27]. However recent explosion in fungal genome sequencing greatly expanded the fungal genomics and molecular diversity of these organisms. Compared to the genome size of animals and plants, the genome sizes of fungi are small [28]. The genome size of model fungi *S. cerevisae* is bit more than 12 Mb (Table 1). From the studied 172 fungal species, only seven species have genome sizes larger than 100 Mb (Table 1). So, the probability of occurrence of larger genomes in fungi is very small. The genome size of *Cenococcum geophilum* (177.57 Mb) is the largest and the genome size of *Hansenula polymorpha* (8.97 Mb) is the smallest from the studied species. Both species belong to Ascomycota. In the group of Basidiomycota species, the genome size of *Wallemia sebi* (9.82 Mb) is the smallest one and genome of *Dendrothele bispora* (130.65) is the largest one (Table 1). No single species from Chytridiomycota, Glomeromycota, Oomycota, Stramenopiles, Mucoromycotina have genome size larger than 100 Mb. Although there is large variation in genome size in fungi, the average genome size of fungal species taken during this study is 42. 30 Mb (Table 1). The average genome sizes of fungal species belonging to different phyla are provided in Table 2. From the table we can observe that the average genome size of Ascomycota group of fungi is 36.91 Mb. The average genome size of Basidiomycota group is 46.48 Mb. The average genome size of Oomycota group of fungi is 74.85 Mb which is the highest among all groups (Table 2). If we consider about the coding gene sequence in fungi, in average the Acomycota, Basidiomycota, Oomycota and Mucoromycotina groups encodes for 11129.45, 15431.51, 24173.33, 13306 no. of genes respectively in their genomes (Table 2).

**Table 1:** **List of genome size (Mb), numbers of coding genes, and average numbers of exons present per fungal species from the different phyla of Kingdom Fungi**

**Sl. No**	**Name of Fungal Species**	**Division**	**Genome Size Mbp**	**No. Of Contigs**	**No. Of Scaffolds**	**No of Gene Models**	**Average Exons Per Gene**
1	*Acidomyces richmondensis*	Ascomycota	29.88	3164	3164	11202	2.28
2	*Acremonium alcalophilum*	Ascomycota	54.42	865	15	9491	4.05
3	*Agaricus bisporus*	Basidiomycota	30.2	254	29	10438	6.05
4	*Amanita muscaria Koide*	Basidiomycota	40.70	3814	1101	18153	4.54
5	*Amorphotheca resinae*	Ascomycota	28.63	261	32	9642	2.97
6	*Anthostoma avocetta*	Ascomycota	56.23	1038	786	15755	2.93
7	*Antrodia sinuosa*	Basidiomycota	30.17	1482	1387	11327	6.09
8	*Apiospora montagnei*	Ascomycota	47.67	706	686	16992	2.52
9	*Aplanochytrium kerguelense*	Stramenopiles	35.77	523	207	11892	2.75
10	*Aplosporella prunicola*	Ascomycota	32.82	763	334	12579	2.67
11	*Ascobolus immersus*	Ascomycota	59.53	1225	706	17877	2.67
12	*Ascoidea rubescens*	Ascomycota	17.50	101	63	6802	1.39
13	*Aspergillus acidus*	Ascomycota	37.47	318	107	13530	3.10
14	*Aspergillus niger*	Ascomycota	34.85	24	24	11910	3.38
15	*Atractiellales sp.*	Basidiomycota	51.47	3076	1998	17606	5.30
16	*Aulographum hederae*	Ascomycota	31.98	613	173	12127	2.66
17	*Aurantiochytrium limacinum*	Stramenopiles	60.93	1118	181	14859	1.45
18	*Aureobasidium pullulans*	Ascomycota	29.62	84	75	10809	2.51
19	*Auricularia subglabra*	Basidiomycota	76.85	2158	761	25459	4.80
20	*Babjeviella inositovora*	Ascomycota	15.22	210	49	6403	1.27
21	*Backusella circina*	Mucoromycotina	48.65	2354	1095	17039	4.25
22	*Baudoinia compniacensis*	Ascomycota	21.88	35	19	10513	2.14
23	*Bjerkandera adusta*	Basidiomycota	42.73	1263	508	15473	5.59
24	*Boletus edulis*	Basidiomycota	46.64	4723	1099	16933	4.88
25	*Botryobasidium botryosum*	Basidiomycota	46.67	1446	334	16526	5.58
26	*Calocera cornea*	Basidiomycota	33.24	1032	545	13177	4.44
27	*Calocera viscosa*	Basidiomycota	29.10	487	214	12378	4.59
28	*Candida caseinolytica*	Ascomycota	9.18	49	6	4657	1.20
29	*Catenaria anguillulae*	Blastocladiomycota	36.22	2577	801	14188	2.50
30	*Cenococcum geophilum*	Ascomycota	177.57	2893	268	27529	4.08
31	*Cercospora zeae-maydis*	Ascomycota	46.61	2555	917	12020	2.32
32	*Chalara longipes*	Ascomycota	52.43	175	54	19765	3.06
33	*Choiromyces venosus*	Ascomycota	126.04	3183	1176	17986	2.84
34	*Cochliobolus sativus*	Ascomycota	34.42	478	157	12250	2.63
35	*Coemansia reversa*	Kickellomycotina	21.84	1063	346	7347	1.51
36	*Conidiobolus coronatus*	Entomophthoromycota	39.90	7809	1050	10635	2.78
37	*Coniophora puteana*	Basdiomycota	42.97	1034	210	13761	6.11
38	*Coprinopsis cinerea*	Basdiomycota	37.5	---	---	---	---
39	*Cortinarius glaucopus*	Basidiomycota	63.45	2103	769	20377	5.05
40	*Cronartium quercuum*	Basidiomycota	76.57	10431	1198	13903	4.35
41	*Cryphonectria parasitica*	Ascomycota	43.9	33	26	11609	2.91
42	*Cryptococcus vishniacii*	Basidiomycota	19.69	137	50	7232	6.25
43	*Cucurbitaria berberidis*	Ascomycota	32.91	184	42	12439	2.71
44	*Cyberlindnera jadinii*	Ascomycota	13.02	392	76	6038	1.35
45	*Cylindrobasidium torrendii*	Basidiomycota	31.57	1222	1149	13940	5.17
46	*Dacryopinax sp.*	Basidiomycota	29.50	878	99	10242	4.83
47	*Daedalea quercina*	Basidiomycota	32.74	1025	357	12199	5.80
48	*Daldinia eschscholzii*	Ascomycota	37.55	512	398	11173	2.89
49	*Dekkera bruxellensis*	Ascomycota	13.37	1374	84	5600	1.44
50	*Dendrothele bispora*	Basidiomycota	130.65	6351	3942	33645	5.09
51	*Dichomitus squalens*	Basidiomycota	42.75	2852	542	12290	5.84
52	*Didymella exigua*	Ascomycota	34.39	1010	176	12394	2.46
53	*Dioszegia cryoxerica*	Basidiomycota	39.52	1318	865	15948	5.36
54	*Dissoconium aciculare*	Ascomycota	26.54	232	54	10299	2.17
55	*Dothidotthia symphoricarpi*	Ascomycota	34.43	268	59	11790	2.71
56	*Eurotium rubrum*	Ascomycota	26.21	371	110	10076	3.07
57	*Exidia glandulosa*	Basidiomycota	78.17	4024	1727	26765	4.83
58	*Exobasidium vaccinii*	Basidiomycota	16.99	246	119	7453	2.79
59	*Fibulorhizoctonia sp.*	Basidiomycota	95.13	3901	1918	32946	4.63
60	*Fomitiporia mediterranea*	Basidiomycota	63.35	5766	1412	11333	6.06
61	*Fomitopsis pinicola*	Basidiomycota	46.30	988	504	13885	5.56
62	*Galerina marginata*	Basidiomycota	59.42	1272	414	21461	5.30
63	*Ganoderma sp.*	Basidiomycota	39.52	503	156	12910	5.82
64	*Gloeophyllum trabeum*	Basidiomycota	37.18	2289	443	11846	6.14
65	*Glomerella acutata*	Ascomycota	50.04	378	307	15777	2.83
66	*Glomerella cingulata*	Ascomycota	58.84	774	119	18975	2.79
67	*Gonapodya prolifera*	Monoblepharidomycetes	48.79	1154	352	13902	5.58
68	*Gymnascella aurantiaca*	Ascomycota	25.35	356	347	9106	3.12
69	*Gymnascella citrina*	Ascomycota	25.16	305	272	9779	2.99
70	*Gyrodon lividus*	Basidiomycota	43.05	1390	369	11779	5.75
71	*Hanseniaspora valbyensis*	Ascomycota	11.46	1163	646	4800	1.20
72	*Hansenula polymorpha*	Ascomycota	8.97	9	7	5177	1.20
73	*Hebeloma cylindrosporum*	Basidiomycota	37.61	222	222	16841	5.05
74	*Heterobasidion annosum*	Basidiomycota	33.7	18	15	13405	5.54
75	*Hydnomerulius pinastri*	Basidiomycota	38.28	2315	603	13270	5.84
76	*Hypholoma sublateritium*	Basidiomycota	48.03	1329	704	17911	5.29
77	*Hyphopichia burtonii*	Ascomycota	12.40	105	27	6002	1.22
78	*Hypoxylon sp.*	Ascomycota	46.59	580	505	12256	2.90
79	*Jaapia argillacea*	Basidiomycota	45.05	1182	295	5.53	5.53
80	*Laccaria amethystina*	Basidiomycota	52.20	4756	1299	21066	4.49
81	*Laccaria bicolor*	Basidiomycota	60.71	584	55	23132	5.28
82	*Laetiporus sulphureus*	Basidiomycota	39.92	1207	403	13774	5.72
83	*Lentinus tigrinus*	Basidiomycota	39.68	571	286	15581	5.59
84	*Leucogyrophana mollusca*	Basidiomycota	35.19	1347	1262	14619	5.89
85	*Lichtheimia hyalospora*	Mucoromycotina	33.28	2294	2222	12062	4.99
86	*Lipomyces starkeyi*	Ascomycota	21.27	439	117	8192	2.85
87	*Lophiostoma macrostomum*	Ascomycota	42.58	1294	1282	16160	2.74
88	*Macrolepiota fuliginosa*	Basidiomycota	46.40	4852	3478	15801	5.39
89	*Melampsora laricis-populina*	Basidiomycota	101.1	---	462	19694	---
90	*Melanconium sp.*	Ascomycota	58.52	465	100	16656	2.68
91	*Melanomma pulvis-pyrius*	Ascomycota	42.09	1771	1754	15881	2.77
92	*Meliniomyces bicolor*	Basidiomycota	82.38	301	206	18619	2.96
93	*Metschnikowia bicuspidata*	Ascomycota	16.06	421	48	5851	1.27
94	*Mixia osmundae*	Basidiomycota	13.63	204	156	6903	4.54
95	*Monascus purpureus*	Ascomycota	23.44	319	296	8918	3.19
96	*Monascus ruber*	Ascomycota	24.80	362	320	9650	3.13
97	*Mortierella elongata*	Mucoromycotina	49.96	3314	473	14964	3.47
98	*Mucor circinelloides*	Mucoromycotina	36.6	26	26	11719	3.8
99	*Myceliophthora thermophila*	Ascomycota	38.74	7	7	9110	2.83
100	*Mycosphaerella graminicola*	Ascomycota	39.7	---	129	10952	---
101	*Myriangium duriaei*	Ascomycota	25.69	32	16	10685	2.37
102	*Nadsonia fulvescens*	Ascomycota	13.75	64	20	5657	1.57
103	*Neolentinus lepideus*	Basidiomycota	35.64	1215	331	13164	5.71
104	*Neurospora discreta*	Ascomycota	37.3	---	176	9948	---
105	*Neurospora tetrasperma*	Ascomycota	37.8	542	155	10640	2.72
106	*Oidiodendron maius*	Ascomycota	46.43	387	100	16703	2.97
107	*Pachysolen tannophilus*	Ascomycota	12.60	583	198	5675	1.33
108	*Patellaria atrata*	Ascomycota	28.69	501	127	9794	2.97
109	*Paxillus rubicundulus*	Basidiomycota	53.01	7170	6945	22065	3.81
110	*Penicillium brevicompactum*	Ascomycota	32.11	96	35	11536	3.09
111	*Penicillium canescens*	Ascomycota	33.26	248	62	12374	3.12
112	*Penicillium janthinellum*	Ascomycota	35.15	273	94	12098	3.07
113	*Penicillium raistrickii*	Ascomycota	31.44	104	76	11368	3.11
114	*Phlebia brevispora*	Basidiomycota	49.96	3178	1645	16170	5.66
115	*Phlebiopsis gigantea*	Basidiomycota	30.14	1195	573	11891	6.00
116	*Phycomyces blakesleeanus*	Mucoromycotina	53.9	350	80	16528	4.5
117	*Phytophthora capsici*	Oomycota	64	10760	917	19805	2.20
118	*Phytophthora cinnamomi*	Oomycota	77.97	9537	1314	26131	2.10
119	*Phytophthora sojae*	Oomycota	82.60	1643	83	26584	2.39
120	*Pichia stipitis*	Ascomycota	15.4	---	394	5841	~1
121	*Piedraia hortae*	Ascomycota	16.95	214	132	7572	1.84
122	*Piloderma croceum*	Basidiomycota	59.33	4469	715	21583	4.75
123	*Piromyces sp.*	Neocallimastigomycota	71.02	17217	1656	14648	3.09
124	*Pisolithus microcarpus*	Basidiomycota	53.03	5476	1064	21064	4.04
125	*Pleomassaria siparia*	Ascomycota	43.18	1023	193	13486	2.81
126	*Pleurotus ostreatus*	Basidiomycota	35.6	3272	572	11603	6.1
127	*Polychaeton citri*	Ascomycota	27.21	451	416	10582	2.12
128	*Polyporus arcularius*	Basidiomycota	43.45	2601	2540	17525	5.27
129	*Punctularia strigosozonata*	Basidiomycota	34.17	1327	195	11538	6.23
130	*Pycnoporus sanguineus*	Basidiomycota	36.04	2046	657	14165	5.59
131	*Ramaria rubella*	Basidiomycota	105.46	5927	1553	19287	5.53
132	*Rhizophagus irregularis*	Glomeromycota	91.08	28405	28371	30282	3.46
133	*Rhizopus microsporus*	Mucoromycotina	25.97	823	131	10905	4.03
134	*Rhodotorula graminis*	Basidiomycota	21.01	620	26	7283	6.24
135	*Rickenella mellea*	Basidiomycota	46.03	1236	1092	18952	4.98
136	*Saccharata proteae*	Ascomycota	31.43	727	245	9234	3.08
137	*Saccharomyces cerevisiae*	Ascomycota	12.07	16	16	6575	1.04
138	*Saitoella complicata*	Ascomycota	14.14	35	35	7034	2.23
139	*Schizophyllum commune Loenen D*	Basidiomycota	35.88	1822	1774	13827	5.55
140	*Schizophyllum commune Tattone D*	Basidiomycota	36.46	1757	1707	15199	5.27
141	*Schizopora paradoxa*	Basidiomycota	44.41	1342	1291	17098	5.78
142	*Scleroderma citrinum*	Basidiomycota	56.14	3919	938	21012	4.33
143	*Sebacina vermifera*	Basidiomycota	38.09	2457	546	15312	4.94
144	*Septoria musiva*	Ascomycota	29.35	706	72	10233	2.44
145	*Serpula lacrymans*	Basidiomycota	42.73	375	36	12789	5.73
146	*Sistotremastrum niveocremeum*	Basidiomycota	35.36	699	179	13080	5.95
147	*Sodiomyces alkalinus*	Ascomycota	43.45	290	25	9411	3.32
148	*Spathaspora passalidarum*	Ascomycota	13.2	26	8	5983	1.2
149	*Sporobolomyces roseus*	Basidiomycota	21.2	---	76	5536	---
150	*Sporormia fimetaria*	Ascomycota	25.89	293	140	10783	2.70
151	*Stereum hirsutum*	Basidiomycota	46.51	995	159	14072	6.52
152	*Suillus brevipes*	Basidiomycota	51.71	4139	1550	22453	4.54
153	*Talaromyces aculeatus*	Ascomycota	37.27	165	49	13793	3.16
154	*Terfezia boudieri*	Ascomycota	63.23	2078	516	10200	3.61
155	*Thermoascus aurantiacus*	Ascomycota	28.49	196	48	8798	3.33
156	*Thielavia appendiculata*	Ascomycota	32.74	501	109	11942	2.77
157	*Thielavia arenaria*	Ascomycota	30.99	354	69	10954	2.80
158	*Thielavia hyrcaniae*	Ascomycota	31.18	972	251	11338	2.73
159	*Trametes versicolor*	Basidiomycota	44.79	1443	283	14296	5.81
160	*Trichaptum abietinum*	Basiodiomycota	40.61	1345	492	14978	5.65
161	*Trichoderma citrinoviride*	Ascomycota	33.48	699	533	9737	3.10
162	*Trypethelium eluteriae*	Ascomycota	32.16	747	730	11858	2.83
163	*Tulasnella calospora*	Basidiomycota	62.39	6848	1335	19659	4.65
164	*Umbelopsis ramanniana*	Mucoromycotina	23.08	239	198	9931	4.75
165	*Wallemia sebi*	Basidiomycota	9.82	114	56	5284	4.03
166	*Wickerhamomyces anomalus*	Ascomycota	14.15	207	46	6423	1.42
167	*Wilcoxina mikolae*	Ascomycota	117.29	5591	1604	13093	3.24
168	*Wolfiporia cocos*	Basidiomycota	50.48	2228	348	12746	6.31
169	*Xanthoria parietina*	Ascomycota	31.90	302	39	10818	2.98
170	*Xylona heveae*	Ascomycota	24.34	56	27	8205	3.41
171	*Zasmidium cellare*	Ascomycota	38.25	365	267	16015	2.50
172	*Zopfia rhizophila*	Ascomycota	152.78	1349	864	21730	2.77
	**Average**		**42.300**			**13437.21**	**3.79**

The fungal classifications (phyla/sub-phyla) are based on reports of Humber (2012) and Hibbet et al. (2007) [22,23].

**Table 2:** **Average genome size, and average number of coding genes and exons present in the different phyla/sub-phyla of the Kingdom Fungi**

**Fungal division**	**Average Genome Size (Mb)**	**Average No. Of Genes**	**Average No. Of Exons**
Ascomycota	36.91	11129.45	2.58
Basidiomycota	46.48	15431.51	5.28
Oomycota	74.85	24173.33	2.23
Mucoromycotina	38.777	13306.85	4.25

**Figure 1:**
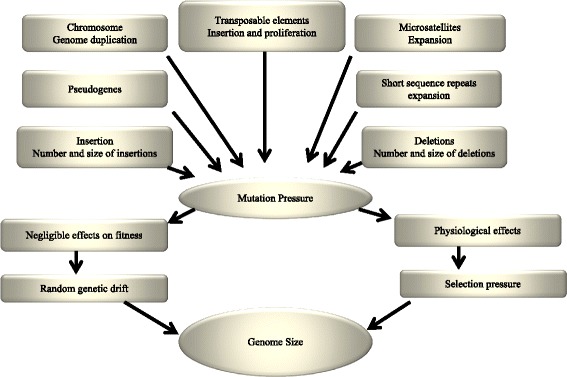
**Role of different forces affecting the evolution of genome size.** The major important factors are transposable elements (TEs), short sequence repeats, microsatellites, genome duplication and others. The mutational and selection pressure plays a significant role in this process. The negligible selective effects governed by genetic drift also contribute for the evolution of genome size. Overall all the forces play a role towards the increase in genome size at different levels. The photograph is adapted according to the report of Petrov [37].

The comparative analysis of fungal genomes show fungi are very divergent [27]. It was earlier thought that genomes of *Magnaporthe grisea* and *Neurospora crassa* share a common ancestor. But, comparative genomes analyses revealed only 47% amino acid sequence identity and absence of conserved synteny [27]. Only few genes are identified to be in conserved co-linearity. This shows that even members of the same genus can show remarkable divergence at the genomic level. A genomic comparison between *Aspergillus nidulans, Aspergillus fumigatus* and *Aspergillus oryzae* shows only 68% of amino acid sequence identity [27]. The genome duplication and translocation have major impact in evolution in yeast (Figure 1) [29,30]. The whole genome duplication in yeast followed by massive gene loss is confirmed by comparative experimental analysis [31,32]. This indicates that fungal genomes are very dynamic in nature. Lavergne et al. [33] reported that genome size reduction can trigger rapid phenotypic evolution in invasive plants. Their report suggests that the invasive genotypes had smaller genomes. Smaller genome sizes have phenotypic effects that increased the invasive potential [33]. But in exception, for example, the duckweeds which are smallest, fast-growing and simplest flowering plants are invasive in nature and contains increased DNA content in their genomes [34].

### Evolution in genome size

Genomes are aggregates of genes and this concept nicely fits with the prokaryotic organisms and viruses [35]. This concept is very inappropriate for eukaryotic organisms as most of the eukaryotic genomes are studded with nongenic and unconstrained repetitive DNA. This can lead to approximately 200,000 fold variation in genome size [36]. The genome size of an organism depends on the particular developmental and ecological need of the organism [37]. The genes are made up of DNA and it is a general assumption that more complex organisms requires more genes and thus contain more DNA in its genomes. The simple organisms probably contain fewer essential genes compared to more complex organisms and thus contain less DNA in its genomes. However this observation is not true. Some very simple organisms could have more DNA content than complex multi-cellular organisms. For example, some amoeba species have 200 times more DNA than humans [38]. Similarly, lilies have 200 times more DNA than that of rice [39]. But in many organisms much of the DNA content is noncoding and repetitive. But it is very important to understand which evolutionary forces produces enormous amount of noncoding DNA? What are the adaptive functions of these nongenic DNA? If these nongenic DNA don’t have any essential adaptive roles, than why natural selection favors the burden of synthesis of extra DNA? Several hypotheses are postulated since long days to address these questions. But still there is debate over it. Some of the hypotheses are discussed later. From the studied fungal genomes, the average genome sizes of Oomycota species (74.85 Mb) are higher than other. The Ascomycota and Mucoromycotina species shares more or less than same average genome size i.e. 36.91 and 37.02 Mb, respectively. In contrary, the average genome sizes of Basidiomycota species is 46.48 Mbs. The increase in genome size in Oomycota species is also directly correlated with the increase in the numbers of average coding gene sequences. The average numbers of coding genes present in Oomycota species are 24173.33 genes per genome which is almost the double number present in Ascomycota and Basidiomycota species.

### The adaptive theories of genome evolution

If certain numbers of genes are responsible for the phenotype and genotypic characters of an organism, why there are extra amounts of DNA in its genome? The adaptive theory explains that this extra DNA abundance is for adaptive function and its content don’t have any significant effects in phenotype of the organism [40]. A large genome directly increases the nuclear and cellular volumes [41]. This largely helps to buffer the fluctuation in the concentration of regulatory proteins or protect coding DNA from spontaneous mutation [42]. So the variation in the genome size is due to adaptive needs or due to natural selection in different organisms [37].

### Junk DNA theory of genome evolution

The junk DNA hypothesis suggests that these extra DNAs are useless, maladaptive DNAs and fixed by random drift [43]. These DNAs are carried in chromosome and don’t have any significant role in the phenotype of an organism [43]. These junk DNAs are known as parasitic DNA or transposable elements (TEs) [44]. The mutational mechanisms of DNA gain or loss can lead to minor changes in the genome of an organism, but changes in genome size may occur by the involvement of different evolutionary forces [37]. An increase in transposition rate certainly can lead to an increase in genome size [37]. Instead of thinking in genome size evolution by adaptive evolution theory or by junk DNA theory, it is very important to understand which evolutionary force is responsible for changes in genome size. The mutational and selective forces might have vast potential to affect the change in genome sizes (Figure 1) [37]. If we can get the specific clue, we can try to estimate the strength of individual force and whether the magnitude of individual force may produce changes in genome sizes. This approach can explain the quantitative sense about genetic mechanism and the selective forces that affect the genome size.

The activities of transposable elements are very fast and can able to amplify the a transposable copy number into 20-100 copies (~0.1-1 Mbp) in a single generation [45,46]. The changes in genome size through spontaneous deletion or insertion are relatively slow [47]. For example, the *Drosophila melanogaster* genome losses less than a single base pair per generation [47]. If there is strong selection in increasing in gnome sizes, strong mutational pressure also can not affect the evolution of genome size [37]. However, strong selection for increase in genome size can substantially slow down the impact of mutation rate. If we can get the information of time scale of genome size divergence, then we can infer the genome-size changes between two closely related organisms. If we will consider the evolutionary development of fungus, Ascomycota has higher evolutionary rate than Basidiomycota [48]. But when we compared the average genome size of Ascomycota, Basidiomycota and Mucoromycotina, we found that the genome size of Basidiomycota is larger than the genome size of Ascomycota and Mucoromycotina. This may suggests that the evolution of fungal genome size is due to addition of nucleotides/DNA contents rather than deletion of nucleotides.

Some forces act on the traits correlated with total genome size of an organism [37]. In this case, natural selection forces affect only to few genomic components. For example, the increase in rate of heterochromatin shrinkage through heterochromatic DNA should not affect the size of euchromatin [37]. Similarly, the expansion in satellite DNA should not hamper the size of satellite free sequences. Another important question is that, whether different genomic components are varying together in a correlated fashion during evolution of genome size? Although there are no significant current evidences regarding this question, there are certain cytogenetic and molecular studies available. The cytogenetic study revealed that genome size differences are scattered throughout the euchromatic portion of the genome [49-52]. Comparison of orthologous introns revealed correlation between average size of intron and genome size [53]. The changes in the intron length do not account for the changes in the genome size. Although transposable elements are largely associated with the increase in genome size, presence of increased simple repeated sequences, pseudogenes, increased size of inter-enhancer spacers and microsatellites are also associated with increase in genome size (Figure 1) [54-56]. When there are changes in genome size, they do it across all the genomic components. This suggests that a global force acts as the direct agent for change in genome size. So, from our study we can speculate that Oomycota species might harbors high densities of TEs, simple repeat sequences, microsatellites and pseudogenes. Similarly, the Basidiomycota species might have more densities of TEs, simple repeat sequences, microsatellites and pseudogenes compared to Ascomycota and other groups. Whitney et al. [57] reported about the nonadaptive process in plant genome size evolution. They hypothesized that genome expansion is maladaptive and lineages with small effective population size evolve larger genomes than those with large population size. In addition, mating systems are likely to affect genome size evolution via population size and spread of transposable elements [57].

## Conclusion

The question of genome size (C-value paradox) is very puzzling. Most probably we can better understand about the evolution of fungal genome size by completely understanding the roles of noncoding DNAs. It is also equally important to understand whether the addition and deletion of additional DNA content varies between species to species and at organism level too. Although experimental approach like cytogenetic study of euchromatic region can give some lime light about this issue, still high fidelity experimental approaches are lacking till to date.

## References

[1] Hawksworth D (2001). The magnitude of fungal diversity: the 1·5 million species estimate revisited. Mycol Res.

[2] Touchon M, Hoede C, Tenaillon O, Barbe V, Baeriswyl S, Bidet P (2009). Organised genome dynamics in the *Escherichia coli* species results in highly diverse adaptive paths. PLoS Genet.

[3] Das M, Royer TV, Leff LG (2007). Diversity of fungi, bacteria, and actinomycetes on leaves decomposing in a stream. Appl Environ Microbiol.

[4] Perotto S, Actis-Perino E, Perugini J, Bonfante P (1996). Molecular diversity of fungi from ericoid mycorrhizal roots. Mol Ecol.

[5] Anderson IC, Campbell CD, Prosser JI (2003). Diversity of fungi in organic soils under a moorland – Scots pine (*Pinus sylvestris* L.) gradient. Environ Microbiol.

[6] Schardl CL, Leuchtmann A, Spiering MJ (2004). Symbioses of grasses with seedborne fungal endophytes. Annu Rev Plant Biol.

[7] Mohanta TK, Bae H (2015). Functional Genomics and Signaling Events in Mycorrhizal Symbiosis. J Plant Interact.

[8] Lau MCY, Jurgens JA, Farrell RL (2009). Correction for pointing et al., highly specialized microbial diversity in hyper-arid polar desert. Proc Natl Acad Sci.

[9] Ma L, Catranis CM, Starmer WT, Rogers SO (1999). Revival and characterization of fungi from ancient polar ice. Mycologist.

[10] Arnold AE, Maynard Z, Gilbert GS, Coley PD, Kursar TA (2000). Are tropical fungal endophytes hyperdiverse?. Ecol Lett.

[11] Ingham RE, Trofymow JA, Ingham ER, Coleman DC (1985). Interactions of bacteria, fungi, and their nematode grazers: effects on nutrient cycling and plant growth. Ecol Monogr.

[12] Yuste JC, Peñuelas J, Estiarte M, Garcia-Mas J, Mattana S, Ogaya R (2011). Drought-resistant fungi control soil organic matter decomposition and its response to temperature. Glob Chang Biol.

[13] Rineau F, Roth D, Shah F, Smits M, Johansson T, Canbäck B (2012). The ectomycorrhizal fungus *Paxillus involutus* converts organic matter in plant litter using a trimmed brown-rot mechanism involving Fenton chemistry. Environ Microbiol.

[14] Barke J, Seipke RF, Grüschow S, Heavens D, Drou N, Bibb MJ (2010). A mixed community of actinomycetes produce multiple antibiotics for the fungus farming ant Acromyrmex octospinosus. BMC Biol.

[15] Whitt J, Shipley SM, Newman DJ, Zuck KM (2014). Tetramic acid analogues produced by co-culture of *Saccharopolyspora erythraea* with *Fusarium pallidoroseum*. J Nat Prod.

[16] Demain A, Martín J-F, García-Estrada C, Zeilinger S (2014). Valuable secondary metabolites from fungi. Biosynth Mol Genet Fungal Second Metab SE - 1.

[17] Montoya S, Sánchez Ó, Levin L: Mathematical Modeling of Lignocellulolytic Enzyme Production from Three Species of White Rot Fungi by Solid-State Fermentation. In Adv Comput Biol SE - 52. Volume 232. Edited by Castillo LF, Cristancho M, Isaza G, Pinzón A, Rodríguez JMC. Springer International Publishing; 2014:371–377.

[18] Anasonye F, Winquist E, Kluczek-Turpeinen B, Räsänen M, Salonen K, Steffen KT (2014). Fungal enzyme production and biodegradation of polychlorinated dibenzo-p-dioxins and dibenzofurans in contaminated sawmill soil. Chemosphere.

[19] Nazari L, Pattori E, Terzi V, Morcia C, Rossi V (2014). Influence of temperature on infection, growth, and mycotoxin production by *Fusarium langsethiae* and *F. sporotrichioides* in durum wheat. Food Microbiol.

[20] Kumara PM, Soujanya KN, Ravikanth G, Vasudeva R, Ganeshaiah KN, Shaanker RU (2014). Rohitukine, a chromone alkaloid and a precursor of flavopiridol, is produced by endophytic fungi isolated from Dysoxylum binectariferum Hook.f and Amoora rohituka (Roxb). Wight &amp. Arn Phytomedicine.

[21] Agarwal V, Moore BS (2014). Fungal polyketide engineering comes of age. Proc Natl Acad Sci.

[22] Hibbett D (2007). A higher level phylogenetic classification of the fungi. Mycol Res.

[23] Humber RA (2012). A new phylum and reclassification for entomophthoroid fungi. Mycotaxon.

[24] Grigoriev IV, Nikitin R, Haridas S, Kuo A, Ohm R, Otillar R (2014). MycoCosm portal: gearing up for 1000 fungal genomes. Nucleic Acids Res.

[25] Nordberg H, Cantor M, Dusheyko S, Hua S, Poliakov A, Shabalov I (2014). The genome portal of the Department of Energy Joint Genome Institute: 2014 updates. Nucleic Acids Res.

[26] Grigoriev IV, Cullen D, Goodwin SB, Hibbett D, Jeffries TW, Kubicek CP (2011). Fungal Biology Fueling the future with fungal genomics. Mycology.

[27] Galagan JE, Henn MR, Ma L-J, Cuomo C, Birren B (2005). Genomics of the fungal kingdom: insights into eukaryotic biology. Genome Res.

[28] Tunlid A, Talbot NJ (2002). Genomics of parasitic and symbiotic fungi. Genomics.

[29] Dunham MJ, Badrane H, Ferea T, Adams J, Brown PO, Rosenzweig F (2002). Characteristic genome rearrangements in experimental evolution of *Saccharomyces cerevisiae*. Proc Natl Acad Sci U S A.

[30] Koszul R, Caburet S, Dujon B, Fischer G (2004). Eucaryotic genome evolution through the spontaneous duplication of large chromosomal segments. EMBO J.

[31] Dujon B, Sherman D, Fischer G, Durrens P, Casaregola S, Lafontaine I (2004). Genome evolution in yeasts. Nature.

[32] Kellis M, Birren BW, Lander ES (2004). Proof and evolutionary analysis of ancient genome duplication in the yeast *Saccharomyces cerevisiae*. Nature.

[33] Lavergne S, Muenke NJ, Molofsky J (2010). Genome size reduction can trigger rapid phenotypic evolution in invasive plants. Ann Bot.

[34] Wang W, Kerstetter R, Michael TP (2011). Evolution of genome size in duckweeds (Lemnaceae). J Bot.

[35] Petrov D (2002). Mutational equilibrium model of genome size evolution. Theor Popul Biol.

[36] Gregory TR, Hebert PDN (1999). The modulation of DNA content: proximate causes and ultimate consequences. Genome Res.

[37] Petrov D (2001). Evolution of genome size: new approaches to an old problem. Trends Genet.

[38] Thomas C (1971). The genetic organization of chromosomes. Annu Rev Genet.

[39] Sahin A, Kaauwen MV, Esselink D, Bargsten JW, Tuyl JM, Visser RG (2012). Generation and analysis of expressed sequence tags in the extreme large genomes *Lilium* and *Tulipa*. BMC Genomics.

[40] Oslon-Manning CF, Wagner MR, Mitchell-Olds T (2013). Adaptive evolution: evaluating empirical support for theoretical prediction. Nat Rev Genet.

[41] Cavalier-Smith T (1978). Nuclear volume control by nucleoskeletal DNA, seletion for cell volume and cell growth rate, and the solution of the DNA C-value Paradox. J Cell Sci.

[42] Vinogradov A (1998). Buffering : a possible passive-homeostasis role for redundant DNA. J Theor Biol.

[43] Ohno S (1972). So much “junk” DNA in our genome. Brookhaven Symp Biol.

[44] Orgel L, Crick F (1980). Selfish DNA: the ultimate parasite. Nature.

[45] Petrov DA, Schutzman JL, Hartl DL, Lozovskaya ER (1995). Diverse transposable elements are mobilized in hybrid dysgenesis in *Drosophila virilis*. Proc Natl Acad Sci.

[46] Kalendar R, Tanskanen J, Immonen S, Nevo E, Schulman a H (2000). Genome evolution of wild barley (Hordeum spontaneum) by BARE-1 retrotransposon dynamics in response to sharp microclimatic divergence. Proc Natl Acad Sci U S A.

[47] Petrov D, Hartl D (1998). High rate of DNA loss in the *Drosophila melanogaster* and *Drosophila virilis* species groups. Mol Biol Evol.

[48] Wang H, Guo S, Huang M, Thorsten L, Wei J (2010). Ascomycota has a faster evolutionary rate and higher species diversity than Basidiomycota. Sci China Life Sci.

[49] Keyl H (1965). A demonstrable local and geometric increase in the chromosomal DNA of *Chironomus*. Experientia.

[50] Labani RM, Elkington TT (1987). Nuclear DNA variation in the genus *Allium* L. (Liliaceae). Heredity (Edinb).

[51] Ohri D (1998). Genome size variation and plant systematics. Ann Bot.

[52] Ohri D, Fritsch RM, Hanelt P (1998). Systemafics and plam evolution of genome size in *Allium* (Alliaceae). Plant Syst Evol.

[53] Moriyama EN, Petrov DA, Hartl DL (1998). Letter to the editor genome size and intron size in *Drosophila*. Mol Biol Evol.

[54] Crollius HR (2000). Characterization and repeat analysis of the compact genome of the freshwater pufferfish *Tetraodon nigroviridis*. Genome Res.

[55] Wessler SR (2006). Transposable elements and the evolution of eukaryotic genomes. Proc Natl Acad Sci U S A.

[56] Ellegren H (2004). Microsatellites: simple sequences with complex evolution. Nat Rev Genet.

[57] Whitney KD, Baack EJ, Hamrick JL, Godt MW, Barringer BC, Bennet MD (2010). A role for nonadaptive process in plant genome size evolution. Proc Natl Acad Sci U S A.

